# Transcriptional Suppression, DNA Methylation, and Histone Deacetylation of the Regulator of G-Protein Signaling 10 (RGS10) Gene in Ovarian Cancer Cells

**DOI:** 10.1371/journal.pone.0060185

**Published:** 2013-03-22

**Authors:** Mourad W. Ali, Ercan Cacan, Yuying Liu, Jennifer Young Pierce, William T. Creasman, Mandi M. Murph, Rajgopal Govindarajan, Scott T. Eblen, Susanna F. Greer, Shelley B. Hooks

**Affiliations:** 1 Department of Pharmaceutical and Biomedical Sciences, University of Georgia, Athens, Georgia, United States of America; 2 Department of Biology, Georgia State University, Atlanta, Georgia, United States of America; 3 Department of Cell and Molecular Pharmacology, Medical University of South Carolina, Charleston, South Carolina, United States of America; 4 Department of Obstetrics and Gynecology, Medical University of South Carolina, Charleston, South Carolina, United States of America; 5 Hollings Cancer Center, Medical University of South Carolina, Charleston, South Carolina, United States of America; University of North Dakota, United States of America

## Abstract

RGS10 regulates ovarian cancer cell growth and survival, and RGS10 expression is suppressed in cell models of ovarian cancer chemoresistance. However, the mechanisms governing RGS10 expression in ovarian cancer are poorly understood. Here we report RGS10 suppression in primary ovarian cancer and CAOV-3 ovarian cancer cells compared to immortalized ovarian surface epithelial (IOSE) cells, and in A2780-AD chemoresistant cells compared to parental A2780 cells. RGS10-1 and RGS10-2 transcripts are expressed in ovarian cancer cells, but only RGS10-1 is suppressed in A2780-AD and CAOV-3 cells, and the RGS10-1 promoter is uniquely enriched in CpG dinucleotides. Pharmacological inhibition of DNA methyl-transferases (DNMTs) increased RGS10 expression, suggesting potential regulation by DNA methylation. Bisulfite sequencing analysis identified a region of the RGS10-1 promoter with significantly enhanced DNA methylation in chemoresistant A2780-AD cells relative to parental A2780 cells. DNA methylation in CAOV-3 and IOSE cells was similar to A2780 cells. More marked differences were observed in histone acetylation of the RGS10-1 promoter. Acetylated histone H3 associated with the RGS10-1 promoter was significantly lower in A2780-AD cells compared to parental cells, with a corresponding increase in histone deacetylase (HDAC) enzyme association. Similarly, acetylated histone levels at the RGS10-1 promoter were markedly lower in CAOV-3 cells compared to IOSE cells, and HDAC1 binding was doubled in CAOV-3 cells. Finally, we show that pharmacological inhibition of DNMT or HDAC enzymes in chemoresistant A2780-AD cells increases RGS10 expression and enhances cisplatin toxicity. These data suggest that histone de-acetylation and DNA methylation correlate with RGS10 suppression and chemoresistance in ovarian cancer. Markers for loss of RGS10 expression may identify cancer cells with unique response to therapeutics.

## Introduction

Cancer cells exploit multiple receptor-mediated growth and survival signaling pathways to evade normal quiescence and cell death responses. Amplification of these pathways is a common mechanism in cancer progression. Activation of G-protein coupled receptors by the ligands lysophosphatidic acid (LPA), endothelin, stromal derived growth factor-1 (SDF1), prostaglandins, and thrombin contribute to the progression of multiple cancers, and drugs that block these receptors are currently in various stages of clinical trials as cancer therapeutics [1]. These GPCRs initiate growth and survival signaling cascades by activating cellular G-proteins. G-protein activity is terminated by regulator of G-protein signaling (RGS) proteins that rapidly deactivate G-proteins and control the strength and duration of GPCR-initiated pathways [2]. RGS proteins that suppress oncogenic signals mediated by GPCR ligands are poised to inhibit cancer growth. Indeed, specific RGS proteins have been shown to suppress receptor-stimulated growth and survival signaling in breast, prostate, and ovarian cancer [3]–[5].

Ovarian cancer is the leading cause of death from gynecological cancers and the fifth most common cause of cancer death in women. Less than 50% of ovarian cancer patients survive five years after their diagnosis [6]. Although ovarian cancer is characterized by a high response rate to chemotherapy, its high mortality rate is largely due to the development of resistance to the first-line chemotherapeutic agents [7]. The majority of patients who initially respond to chemotherapy will relapse with chemoresistant disease within two years [8]. Understanding the molecular and genetic changes that drive ovarian cancer progression and the development of acquired chemoresistance may lead to strategies to predict and prevent the occurrence of refractory disease.

We have shown that endogenous RGS proteins suppress ovarian cancer cell growth, migration, and MAP kinase activation in response to LPA, a major autocrine growth factor in ovarian cancer [3],[9]. More recently, we have identified RGS10 as an important regulator of cell survival and chemoresistance. RGS10 transcript expression is downregulated in multiple models of acquired chemoresistance in ovarian cancer, and RGS10 expression levels alter ovarian cancer cell sensitivity to cisplatin and taxane cytotoxicity [10]. These observations suggest that suppression of RGS10 expression may contribute to ovarian cancer progression and the development of chemoresistance by amplifying GPCR-mediated growth and survival signaling pathways. However, the mechanism of suppression of RGS10 expression in ovarian cancer has not been established.

RGS protein expression is dynamically regulated in neural and cardiovascular systems [11] and in cancer progression [12], allowing for complex control over GPCR signaling pathways. Transcriptional and post-translational mechanisms for control of RGS expression are well defined [13]–[16], while epigenetic control of RGS expression by covalent modifications to DNA or histones has been largely unexplored. Gene silencing by DNA methylation and histone deacetylation is an established mechanism in progression of many cancers [17], including ovarian cancer [18]–[20]. The addition of methyl groups to CpG dinucleotides by DNA methyl transferase (DNMT) enzymes and the removal of acetyl groups on lysine residues in histone proteins by histone deacetylase (HDAC) enzymes coordinately suppress transcriptional activity [21]. DNA methylation and DNMT expression increase in ovarian cancer progression [22], and histone deacetylases (HDACs) are also overexpressed in ovarian cancer tissues [23]. This suggests that epigenetic regulation of RGS genes may also contribute to their dynamic expression in cancer progression.

In the current study, we investigated the epigenetic regulation of RGS10 expression in ovarian cancer cells. We focus on two models of RGS10 suppression – CAOV-3 ovarian cancer cells compared to benign ovarian epithelial cells, and chemoresistant A2780-AD cells and their chemosensitive parental cells. We identify significant increases in DNA methylation in chemoresistant cells, and marked decreases in histone acetylation and increases in HDAC1 association at the RGS10 promoter in both CAOV-3 and A2780-AD cells. Our results suggest that epigenetic histone modifications may contribute to the loss of RGS10 expression in ovarian cancer cells, and that DNA methylation may contribute to further loss of expression during acquired chemoresistance.

## Experimental Procedures

### Cells And Reagents

CAOV-3 and SKOV-3 cells were purchased from American Type Culture Collection (ATCC) and maintained in Dulbecco's Modified Eagle's Medium (ATCC) and McCoy's 5A medium (Mediatech, Inc.), respectively, supplemented with 10% FBS (PAA Laboratories, Inc.). The chemosensitive A2780 parental cell line and its multi-drug resistant daughter counterpart A2780-AD cells (derived as described [24]) were generously provided by Dr. Bob Brown, Imperial College London. These cells were maintained in RPMI 1640 medium (ATCC) supplemented with 10% FBS and 5 mM L-glutamine. Chemoresistant cells were further maintained in 1.5 µM cisplatin. Immortalized ovarian surface epithelial cells (IOSE-80, [25]) were generously provided by Dr. Nelly Auersperg (University of British Columbia) and maintained in Media 199: MCDB 105 (1∶1) supplemented with 15% FBS. All cells were grown in 5 mM penicillin-streptomycin at 37°C with 5% carbon dioxide.

5-Aza-2′-deoxycytidine and cisplatin were purchased from Sigma-Aldrich (St. Louis, MO). Antibodies recognizing histone H3 and acetylated histone H3 were from Millipore (Lake Placid, NY). Antibody recognizing histone H3 (acetyl K18) was from Abcam (Cambridge, MA). Antibodies recognizing RGS10 and HDAC1 were obtained from Santa Cruz (Santa Cruz, CA).

### Cellular Viability Assays

1×10^4^ A2780 or A2780-AD cells were seeded in triplicate in 96-well plates and allowed to attach for 24 hours prior to treatment with the indicated concentrations of cisplatin for 48 hours. Cell viability assay was conducted in serum free media containing CellTiter-Blue® reagent (Promega Corporation) as previously described [10].

### Quantitative Real-Time Pcr

mRNA was isolated using Trizol reagent (Invitrogen) and cDNA was synthesized from 2 μg of total RNA using the High Capacity Reverse Transcriptase cDNA kit (Applied Biosystems/Life Technologies). Quantitative real-time polymerase chain reaction was performed using Superscript III kit for RT-PCR (Invitrogen) and Power SYBR Green reagent (Applied Biosystems). Reactions were normalized using the housekeeping gene GAPDH and calculations were performed according to the 2^−ddCT^ method. Fold change in expression was determined in triplicate in three independent experiments, and experimental replicates were tested for significant differences between groups using paired T-tests. Primers used were based on algorithm-generated sequences from Primer Bank (http://pga.mgh.harvard.edu/primerbank/). RGS10 Forward: GAC CCA GAA GGC GTG AAA AGA, RGS10 Reverse: GCT GGA CAG AAA GGT CAT GTA GA, RGS10 variant-1 Forward: CCC GCG GCG ATG TTC AAC C, RGS10-variant-1 Reverse: CTC CAG GGA TGC CGC CCA TT, RGS10-variant-2 Forward: TGC GTG GAA CTT CTC AGG TGG ACA, RGS10 variant-2 Reverse: CCG CCC ATT TGG CTG TGC TCT, RGS2 Forward: AAG ATT GGA AGA CCC GTT TGA G, RGS2 Reverse: GCA AGA CCA TAT TTG CTG GCT, RGS5 Forward: CCC ACT CAT GCC TGG AAA GG, RGS5 Reverse: CTT GGC TGG TTT CTC TGG CT, GAPDH Forward: GCC AAG GTC ATC CAT GAC AAC T, GAPDH Reverse: GAG GGG CCA TCC ACA GTC TT.

To determine the effect of 5-Aza-2′-deoxycytidine exposure on RGS transcript expression, 7×10^5^ SKOV-3 cells were plated in 100 mm tissue culture plates and allowed to attach overnight. The following day, media was aspirated and replaced with 20 μM 5-Aza-2′-deoxycytidine in DMSO or DMSO vehicle control. After 3, 5, 7 and 9 days of drug incubation, the media was aspirated and 7 mL Trizol reagent (Invitrogen) was added. RNA isolation, DNA synthesis, and qRT-PCR were performed as above.

### Isolation Of Ovarian Cancer Cells From Peritoneal Ascites

Peritoneal ascites from ovarian cancer patients at the Medical University of South Carolina (MUSC) were obtained under MUSC Institutional Review Board (IRB) protocol #18983, which included a review of the ethics of the study and specifically approved the study. This protocol involves the use of de-identified human samples for the study of expression and modification of proteins involved in cell signaling and drug resistance in primary ovarian cancer cells. Removal of peritoneal ascites is a standard of care for ovarian cancer patients and the ascites is normally discarded. All samples received were de-identified prior to delivery to laboratory personnel. Patients were informed of the option to participate in the study and verbal consent was obtained by the physician. Written consent was deemed a risk to patient confidentiality by the IRB since signing a consent form for permission to use a de-identified sample would be the only record of patient identity and participation, and therefore the only risk of a breach in patient confidentiality. A record of samples received in the laboratory was logged only by the date of collection. Removal of peritoneal ascites is a standard of care for ovarian cancer patients. No patient identifying information was obtained by researchers in the laboratory. Peritoneal ascites were centrifuged at 1000 rpm for 10 minutes at room temperature and the cell pellets were washed with phosphate buffered saline (PBS). Red blood cells were lysed in RBC lysis buffer (eBioscience, San Diego, CA) for 5 min at room temperature. Lysis buffer was diluted with PBS, the cells centrifuged as above and resuspended in RPMI medium with 10% FBS. The cells were incubated for 1 hr at 37°C with 5% CO2 to allow attachment of fibroblasts and macrophages. Unattached epithelial cells were removed and incubated separately in complete RPMI medium containing 10% fetal bovine serum.

### Rgs10 Immunoblots

To evaluate RGS10 expression in primary ascites and IOSE cells, cell lysates were generated in RIPA buffer (50 mM Tris pH 7.4, 10% glycerol, 150 mM NaCl, 1% Triton X100, 0.5% SDS, 0.5 mM EDTA, 0.5 mM EGTA, 5 mM Na4P2O7, 40 mM β-glycerophosphate, 50 mM NaF, 2 mM phenylmethylsulfonyl fluoride and aprotinin). After sonication and centrifugation, equal amounts of soluble protein were run on a 10–12% SDS PAGE gel, transferred to nitrocellulose, and immunoblotted with RGS10 antibody. To evaluate RGS10 expression in cell lines, 10^5^ cells were lysed in SDS-PAGE sample buffer. The lysates were boiled for five minutes and analyzed using SDS-PAGE. Membranes were incubated with RGS10 primary antibodies (Santa Cruz Biotechnology, Inc.) and HRP-conjugated rabbit secondary antibodies (Pierce) and visualized using ECL reagents (Pierce). Membranes were subsequently blotted with GAPDH antibodies (Life Technologies) as a loading control.

### Bisulfite Sequencing

The Methprimer website [26] (http://www.urogene.org/methprimer/index1.html) was used to analyze CpG content of RGS promoters and to design primers targeting different regions in the RGS10-1 promoter. Four different primer pairs were designed, RGS10-BS1, RGS10-BS2, RGS10-BS3 and RGS10-BS4: RGS10-BS1forward: AAG AAA ATG GGG GTT AAT GAT ATT T, RGS10-BS1reverse: TAC CTC TAA CAA AAC CTT CAA ACT C, RGS10-BS1 amplification region: −121,303,236 to –121,303,086. RGS10-BS2forward: TGT TTT TAA AGT TAG AGA AGT GTT T, RGS10-BS2reverse: CAC AAA CTA AAA AAC CTA AAC CTC, RGS10-BS2 amplification region: –121,303,076 to –121,302,726. RGS10-BS3forward: GAG GAG GTA AAG GTT ATA GGT TGG, RGS10-BS3reverse: AAA TAC ACT AAC CCA AAA AAA ACC CC, RGS10-BS3 amplification region: –121,302,800 to –121,302,514. RGS10-BS4forward: GTT TGG TTA GGA GGA GG, RGS10-BS4reverse: CTC CAA TCT AAA AAA TAC CAC, RGS10-BS4 amplification region: –121,302,327 to –121,301,988.

Genomic DNA was harvested from cells and bisulfite-converted using EZ DNA Methylation-Direct Kit (Zymo Research Corp). ZymoTaq™ DNA polymerase (Zymo Research Corp) was used to amplify different regions in RGS10 promoter of bisulfite-treated genomic DNA and PCR products were analyzed with 2.5% DNA-agarose gels and purified using PureLink Quick Gel Extraction and PCR Purification Combo Kit (Invitrogen). The purified products were ligated in plasmids using StrataClone PCR Cloning Kit (Agilent Technologies) which were then transformed into competent bacteria. 20 individual colonies were isolated from Carbenicillin LB-agar plates and expanded. QIAprep Spin Miniprep Kit (Qiagen Sample & Assay Technologies) was used to purify the plasmids from each colony, which were then sent for sequencing using T7 and/or T3 promoter sequencing primers at UGA Genomics Facility. Clone sequences were subjected to screens for quality and complete conversion, and aligned to genomic RGS10 promoter DNA using BiQ Analyzer software [27].

### Chromatin Immunoprecipitation (chip) Assay

Cells were plated at a density of 2.5×10^6^ in 15 cm-tissue culture plates and crosslinked with 1% formaldehyde for 8 minutes at room temperature. The crosslinking reaction was stopped by the addition of 0.125 M glycine for five minutes at room temperature. Cell nuclei were isolated and concentrated by lysing in fresh SDS lysis buffer (1% SDS, 10 mM EDTA, 50 mM Tris pH 8.0, dH_2_O) plus protease inhibitors for 25 minutes on ice followed by flash freezing in liquid nitrogen. Nuclei were sonicated using a Bioruptor water bath sonicator for 30 sec “On”, 30 sec “Off” 3X to generate an average of 500 bp of sheared DNA. DNA shearing was confirmed by subjecting lysates to 1% agarose gel electrophoresis and visualization by SYBR safe staining. Sonicated lysates were then precleared with salmon-sperm/agarose beads (Upstate) and 5% of the total lysate was stored as input for normalization. Half of the remaining lysate was immunoprecipitated with 5 μg of indicated antibody overnight at 4°C and the other half was immunoprecipitated with control antibody. Following an additional two hour immunoprecipitation with 60 μl of salmon-sperm coated agarose beads, all samples were washed with each of the following buffers: low salt buffer (0.1% SDS, 1% Triton X-100, 2 mM EDTA, 20 mM Tris pH 8.0, 150 mM NaCl, dH2O), high salt buffer (0.1% SDS, 1% Triton X-100, 2 mM EDTA, 20 mM Tris pH 8.0, 500 mM NaCl, dH2O), LiCl (0.25M LiCl, 1% NP40, 1% DOC, 1 mM EDTA, 10 mM Tris pH 8.0, dH2O), and 1xTE. DNA was eluted with SDS elution buffer (1% SDS, 0.1 M NaHCO3, dH2O). Following elution, cross-links were reversed overnight with 5 M NaCl at 65°C and immunoprecipitated DNA was isolated using phenol:chloroform:isopropanol mix (Invitrogen) as per the manufacturer's instructions. Isolated DNA was quantified by Real time PCR on an ABI prism 7900 (Applied Biosystems, Foster City, CA) using the following primers and probe for RGS10: forward, 5′-GGA ACC GCG AGT CCT CAC-3′, reverse, 5′-CCC GGA GCT CTA GGT CCC-3′ and probe, 5′-TGG CTA GGA GGA GGG CGG CG-3′; and for GAPDH: forward, 5′-AAT GAA TGG GCA GCC GTT A-3′, reverse, 5′-TAG CCT CGC TCC ACC TGA CT-3′ and probe, 5′-CCT GCC GGT GAC TAA CCC TGC GCT CCT-3′. Values generated from Real time PCR reactions were calculated based on standard curves generated, were run in triplicate reactions, and were analyzed using the SDS 2.0 program.

## Results

### Suppression Of Rgs10 Expression In Ovarian Cancer Cells

**Figure 1:**
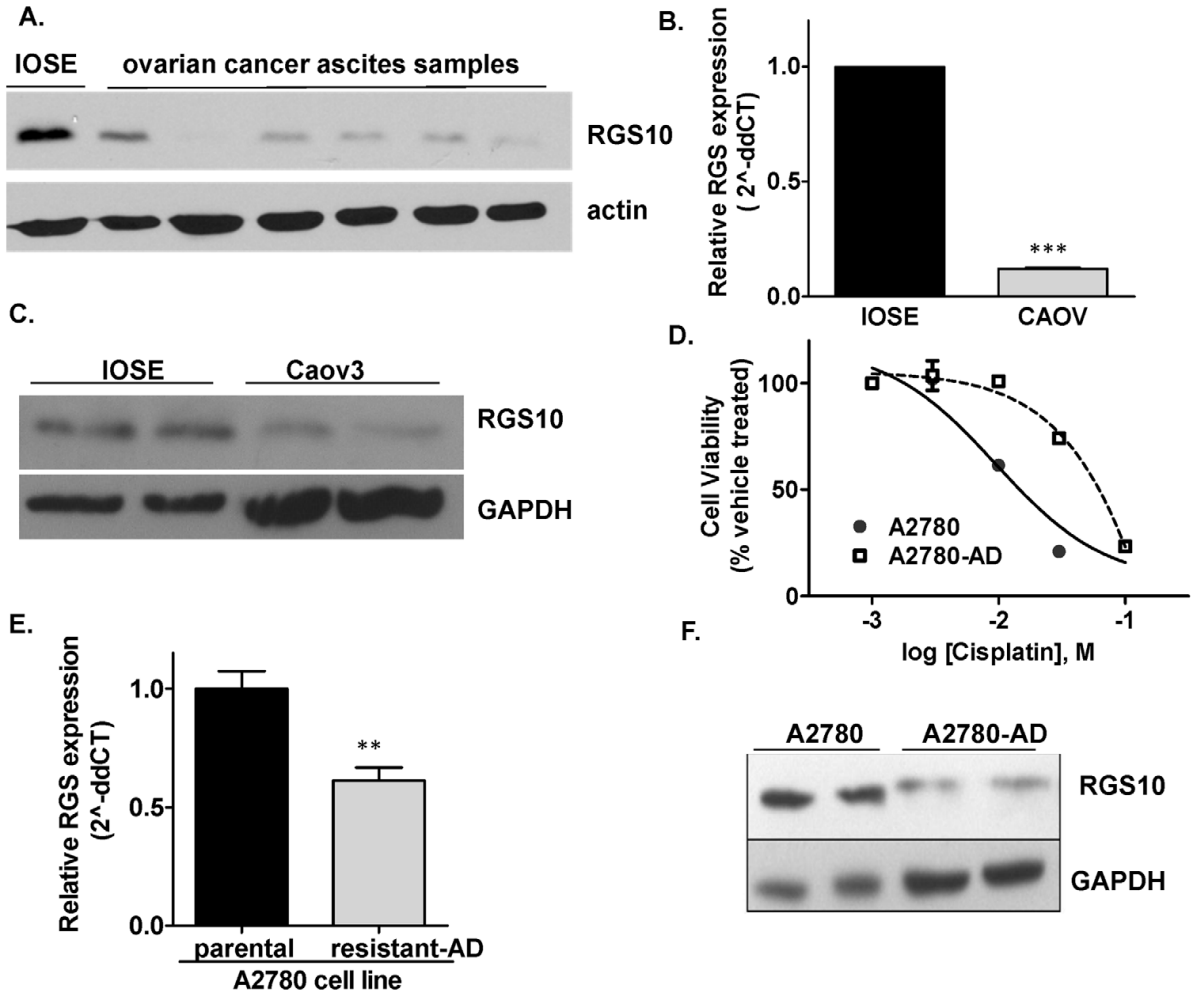
Loss of RGS10 expression in ovarian cancer cells. **A.** Ovarian cancer cells were isolated from patient malignant ascites and RGS10 expression levels were compared to IOSE cells via western blotting. **B.–C.** RGS10 transcript (B) and protein (C) expression levels were compared in CAOV-3 ovarian cancer cell lines and IOSE benign ovarian epithelial cells using qRT-PCR and western blotting. **D.** Cisplatin dose response curves were determined using CellTiter-Blue viability assays in A2780 and A2780-AD cells. **E.–F.** RGS10 transcript (E) and protein (F) levels were compared in chemoresistant A2780-AD cells relative to their parental chemosensitive cell line A2780. **: p<0.01, ***: p<0.0001.

Our previous data demonstrated downregulation of RGS10 transcripts in ovarian cancer cell lines with acquired chemoresistance [10]. To determine if RGS10 is also downregulated in primary ovarian cancer cells, we immunoblotted lysates from the benign, immortalized IOSE cell and from six primary epithelial ovarian cancer cell samples isolated from patient ascites (Figure 1A). RGS10 protein expression was markedly lower in cells from each patient, suggesting that RGS10 expression is suppressed in clinical ovarian cancer. Since patient samples are heterogeneous and non-renewable, their use in defining mechanisms of suppression is limited. To establish a renewable, homogeneous cell model of the loss of RGS10 expression in ovarian cancer, we compared RGS10 expression in IOSE cells and the serous epithelial ovarian cancer cell line CAOV-3 (Figure 1B, C). RGS10 transcript and protein was significantly lower in CAOV-3 cells compared to IOSE control cells.

Our previous observation that RGS10 is suppressed in chemoresistant cells was made in published transcript expression datasets from chemosensitive and chemoresistant ovarian cancer cell pairs [10]. For the current study, we obtained A2780 ovarian cancer cells and their multi-drug resistant derivative A2780-AD. A2780-AD cells were derived from parental A2780 cells via chronic exposure to low-dose cytotoxic drug, and thus represent a model for acquired chemoresistance [28],[29]. We confirmed the loss of sensitivity to cisplatin-induced cytotoxicity in A2780-AD cells, and demonstrated that RGS10 transcript and protein expression is reduced in A2780-AD cells compared to parental A2780 cells (Figure 1 D–F). Taken together, RGS10 transcript and protein expression is reduced in primary ovarian cancer cells and the CAOV-3 cancer cell line relative to immortalized ovarian epithelial cells, and in A2780 cells relative to parental cells. We focused the following studies on these two comparisons.

### Rgs10 Promoters

**Figure 2:**
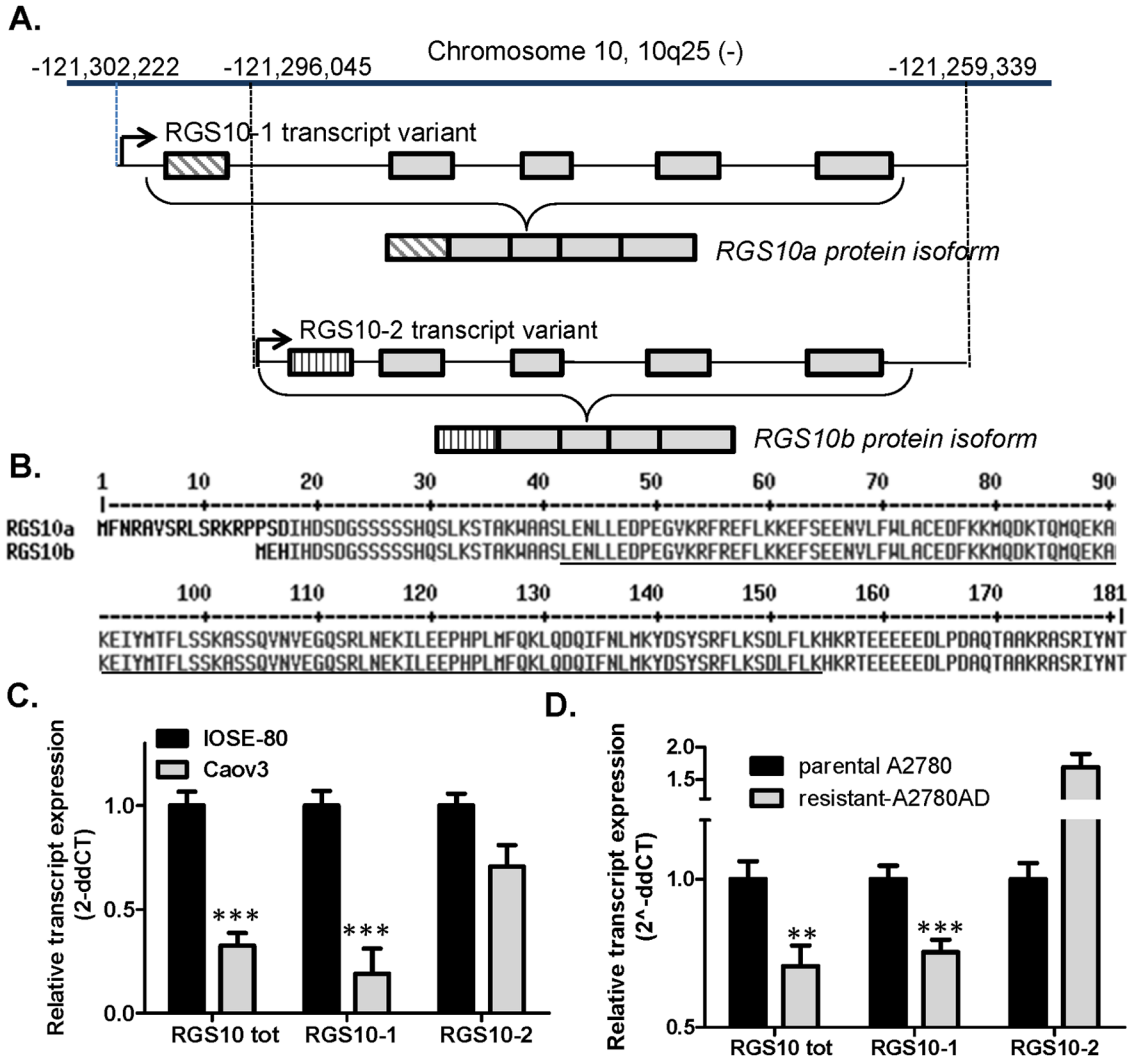
RGS10 gene structure. **A.** The RGS10 gene (geneID: 6001) is located on the negative strand of chromosome 10 (NCBI accession: NC_000010.10) at position −121,302,222 to −121,259,339. Two transcription variants RGS10-1 (accession: NM_001005339) and RGS10-2 (accession: NM_002925) have been reported for RGS10 based on alternate start sites that result in distinct first exons. **B.** The resulting protein isoforms RGS10a (accession: NP_001005339) and RGS10b (accession: NP_002916) vary by only the first 18 or three amino acids. The conserved RGS domain is underlined. **C.–D.** The expression of total RGS10 transcript (RGStot), RGS10-1, and RGS10-2 were determined in IOSE and CAOV-3 cells (C) and in parental A2780 cells and chemoresistant A2780-AD cells (D). **: p<0.01, ***: p<0.0001.

The human RGS10 gene resides on the negative strand of chromosome 10 and contains two transcriptional start sites, giving rise to two distinct transcripts and gene products (Figure 2A, B). The variants have unique first exons, and share four common exons. The longer transcript RGS10-1 gives rise to a 21 kDa protein RGS10a containing 181 amino acids. The shorter transcript variant RGS10-2 gives rise to a 19.5 kDa protein RGS10b comprised of 167 amino acids. Only a single RGS10 immunoreactive band is detectable in ovarian cells, and is consistent with the predicted molecular weight of RGS10a (Figure 1). To determine if both transcripts are detectable and similarly suppressed in ovarian cancer, we performed qRT-PCR using variant-specific primers. Both the long and short transcripts were detected in all cell lines by qRT-PCR, but RGS10-2 was expressed at much lower levels than RGS10-1. RGS10-1 transcript expression in CAOV-3 ovarian cancer cells is approximately 20% of the expression level seen in IOSE cells, comparable to the fold reduction observed for total RGS10 transcript. However, the shorter transcript, RGS10-2, is not significantly different between the two cell lines (Figure 2C). Further, RGS10-1 transcript expression was downregulated in the chemoresistant A2780-AD derivative cell line, while RGS10-2 levels were increased (Figure 2D). These results suggest that suppression of RGS10 transcript in CAOV-3 and A2780-AD ovarian cancer cells is unique to RGS10-1, and suggests that the mechanism may be targeted to the unique promoter region.

### Dna Methylation Of Rgs10 Promoters In Ovarian Cancer Cells

**Figure 3:**
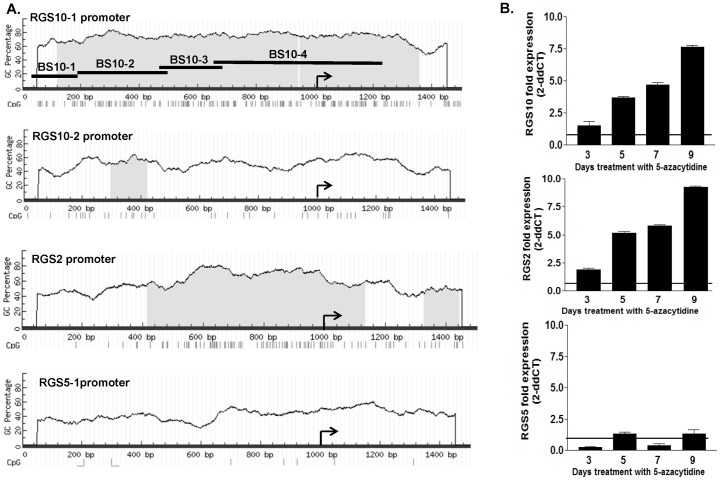
Regulation of RGS genes by DNA methylation. **A.** The promoter regions of RGS10-1, RGS10-2, RGS2, and RGS5 were analyzed for CpG content using the website Methprimer. For each promoter, a region of genomic DNA 1000 basepairs 5′ of the transcriptional start site and 500 basepairs 3′ of the start site were evaluated for percent GC content and individual CpG dinucleotides. Nucleotide position is indicated along the x-axis and GC content is graphed on the y-axis; CpG islands are indicated with shading. Each CpG dinucleotide is indicated by a hash mark below the nucleotide numbering, and the transcriptional start site is indicated with an arrow. Amplification regions for four bisulfite sequencing primer pairs are indicated by horizontal bars (BS10-1, BS10-2, BS10-3, BS10-4). **B.** SKOV-3 cells were treated with vehicle or the DNMT inhibitor 5-Aza for nine days, and the transcript levels of the indicated RGS and GAPDH controls were measured at 3, 5, 7, and 9 days of treatment. The RGS transcript was normalized to GAPDH, and is graphed relative to expression in vehicle treated controls at each time point.

Promoters containing G–C rich “CpG islands” typically have low levels of methylation in normal tissues, but become hypermethylated during cancer progression [30],[31], suggesting that genes with CpG islands in their promoters are potential targets for transcriptional silencing by promoter DNA methylation in cancer cells. Analysis of a region 1 kilobase upstream of the transcriptional start sites and 0.5 kilobase downstream of the start sites of the RGS10-1 and RGS10-2 transcripts reveals a striking difference in the CG content and number of CpG dinucleotides between the two RGS10 promoter regions (Figure 3A). The promoter region of RGS10-1 contains 60–80% CG content and includes approximately 120 CpG dinucleotides, while the RGS10-2 promoter contains less than 30. In comparison, analysis of the RGS2 promoter has CpG content similar to RGS10-1, while the promoter of RGS5 contains few CpG dinucleotides.

The high concentration of CpG dinucleotides in the RGS10-1 promoter suggests that the RGS10 gene is a potential target for regulation by DNMT enzymes and may be suppressed in ovarian cancer progression by enhanced DNA methylation. To test this prediction, we determined the effect of inhibiting DNA methylation on RGS10 expression. The DNMT inhibitor 5-Aza 2′deoxycytidine (5-Aza) blocks the addition of methyl groups to CpG dinucleotides in newly synthesized DNA of proliferating cells [32]. Thus, the effects of 5-Aza on DNA methylation and gene expression are manifest after multiple rounds of cell division. Cells were treated with vehicle or 5-Aza for a total of nine days, and the effect on the transcript levels of RGS10-1, RGS2 and RGS5 was determined every two days. Consistent with CpG island predictions, RGS5 expression does not change with 5-Aza treatment, while RGS10-1 and RGS2 transcript levels are approximately 8-fold higher in 5-Aza treated cells compared to vehicle treated cells (Figure 3B). This result suggests that DNMT enzymes likely contribute to suppression of RGS10-1 transcript levels.

### Bisulfite Sequencing Of Rgs10-1 Promoters

**Figure 4:**
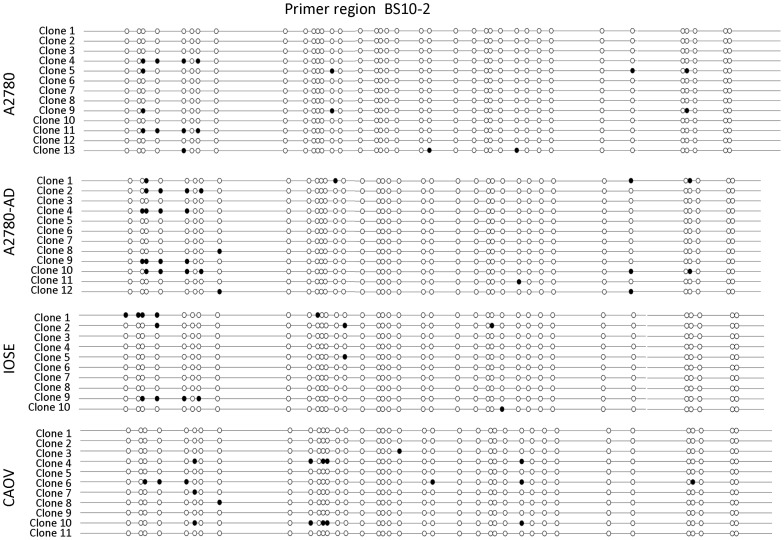
Bisulfite sequencing of RGS10-1 promoter. RGS10 promoter genomic DNA was aligned with individual sequences of cloned PCR products from primer pair BS10-2 amplification of bisulfite treated genomic DNA from the indicated cell lines. Sequences were subjected to quality control analysis and aligned using BiQ Analyzer software. In this conventional ‘lollipop’ representation, each CpG site in the region (−121,303,076 → −121,302,726) is indicated with a circle; filled circles are methylated, unfilled circles are unmethylated. Lollipop representations of the methylation status of each CpG site in RGS10-1 promoter regions amplified by BS10-1, BS10-3, and BS10-4 primer sets are available in the Supporting Information.

We further predicted that the frequency of methylation in RGS10-1 promoters would be higher in ovarian cancer cells with lower RGS10-1 expression levels. Methylated and un-methylated cytosine residues are distinguishable by treatment with bisulfite, which converts unmethylated, but not methylated, cytosine bases to uracil. We first performed bisulfite sequencing to compare the frequency of DNA methylation at RGS10-1 promoters between parental A2780 and A2780-AD chemoresistant cells. Bisulfite-treated genomic DNA was amplified using four overlapping primer pairs designed to fully cover a region from 1000 base pairs upstream to 200 base pairs downstream of the RGS10-1 transcriptional start site. Isolated clones of bisulfite treated genomic DNA were sequenced and aligned to genomic DNA using BiQ Analyzer software to determine the methylation status of each CpG site in the RGS10-1 promoter in at least 10 clones. The results obtained with primer pair BS10-2 are shown in Figure 4, and the results obtained using BS10-1, BS10-3, and BS10-4 are shown in Figure S1.

**Figure 5:**
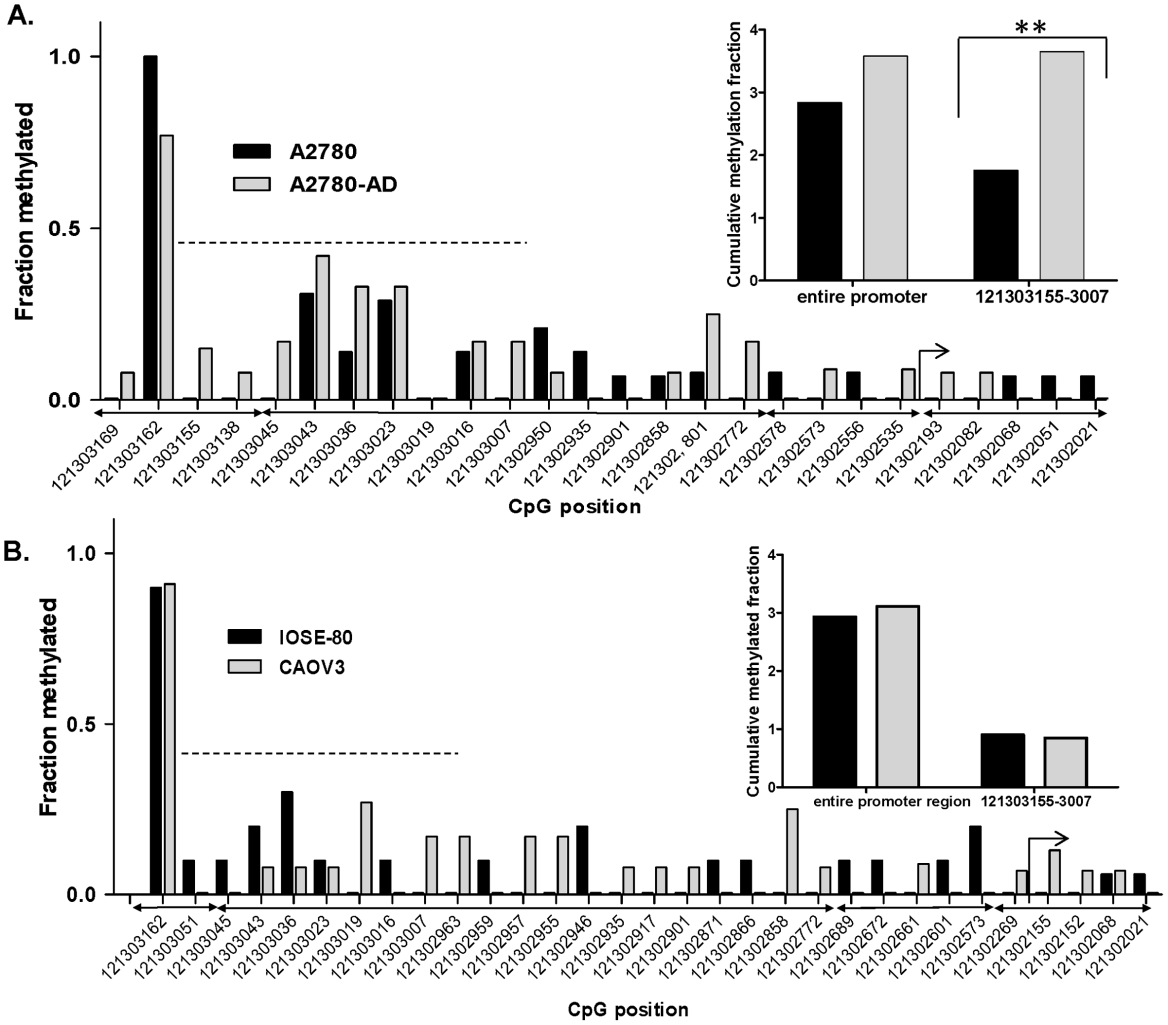
Methylated fraction of CpG dinucleotides across the RGS10-1 promoter in ovarian cell lines. The fraction of clones that were methylated at individual CpG dinucleotides across the RGS10-1 promoter is shown. CpG dinucleotides are labeled by their position on chromosome 10(-). Nucleotides that were not methylated in either cell line are not shown. The complete data set with ratios of sequenced clones is shown in Supplementary Table 1. **A.** Methylation rates are compared between A2780 and A2780-AD cells. **B.** Methylation rates are compared between IOSE and CAOV-3 cells. *Dotted horizontal bar*: region −121,303,155 → −121,303,007. *Insets*: The cumulative fraction of DNA methylation is shown for the entire RGS10-1 promoter and for the indicated region. **: p<0.01. *Bent arrow*: transcriptional start site. *Arrows below x-axis*: sites contained within each primer pair (left to right: BS10-1, BS10-2, BS10-3, BS10-4).

Using the bisulfite sequencing data, we determined the frequency of methylation of CpG sites across the RGS10-1 promoter in A2780 and A2780-AD cells (Figure 5, Table S1). The frequency of methylation at CpG sites across the RGS10-1 promoter was low; the majority of CpG sites were completely unmethylated or were methylated in 10–20% of clones. An exception was the dinucleotide at position −121,030,162, which was highly methylated in both cell lines. Over the entire promoter, the rate of methylation was slightly higher in A2780-AD cells than in parental A2780 cells (Figure 5A, inset). This difference was more pronounced in multiple adjacent CpG sites in region −121,303,155 → −121,303,007 (indicated by dotted horizontal bar, Figure 5A). The overall rate of methylation across this region was doubled in the chemoresistant cells compared to parental cells (Figure 5A, inset). These data suggest that local enhanced DNA methylation in a region approximately 800 basepairs upstream of the transcriptional start site correlates with loss of RGS10 expression in acquired chemoresistance. We next performed the same analysis on RGS10-1 promoters in IOSE and CAOV-3 cells. Methylation rates across the entire RGS10-1 promoter and in the region identified above were similar in IOSE and CAOV-3 cells (Figure 5B). Thus, enhanced DNA methylation of the RGS10-1 promoter does not account for transcriptional suppression in CAOV-3 cells, but was specific to A2780-AD cells.

### Histone Modifications At Rgs10 Promoters In Ovarian Cancer Cells

**Figure 6:**
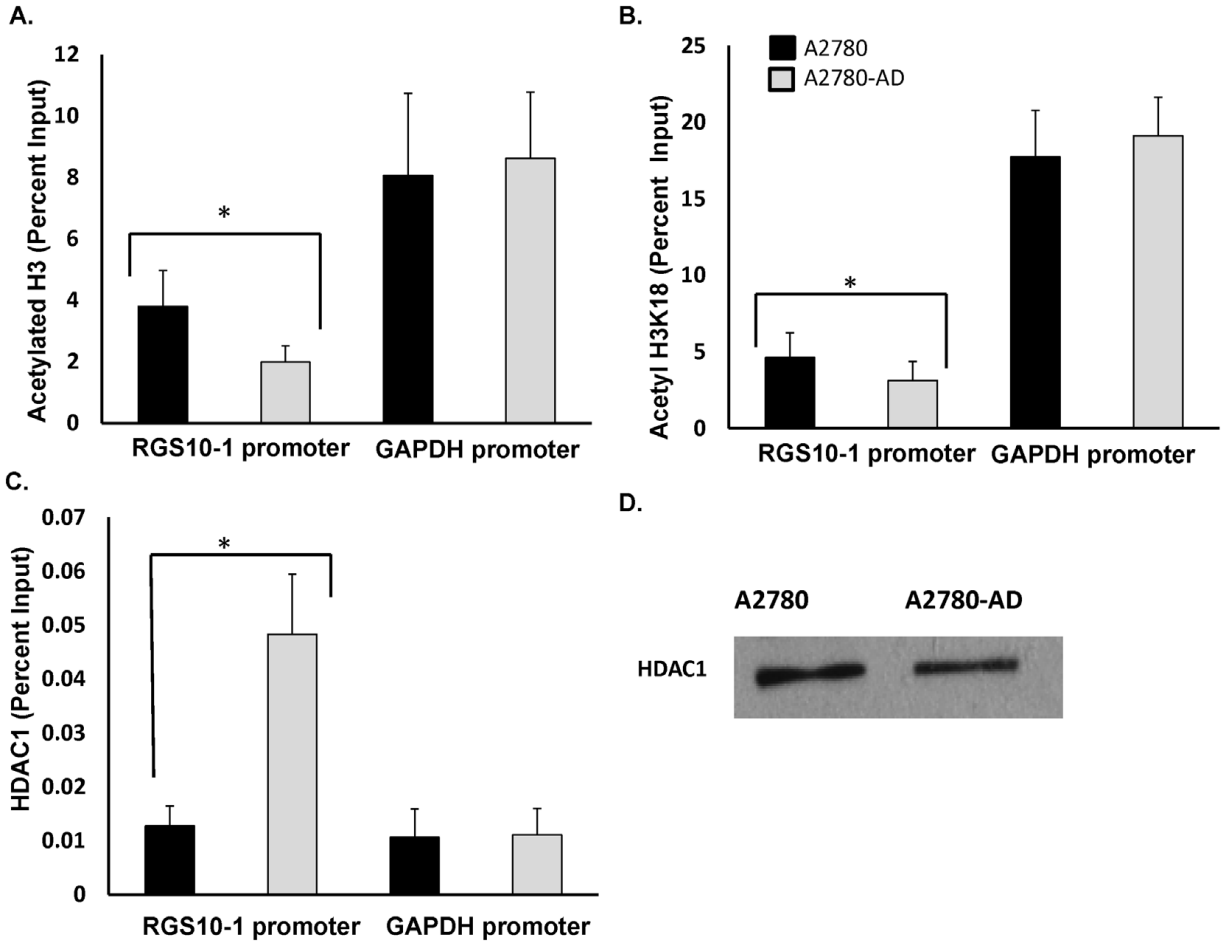
Histone acetylation and HDAC binding at RGS10-1 promoters in chemoresistant A2780-AD cells and parental A2780 cells. ChIP assays were carried out in A2780 parental cells and multi-drug resistant A2780-AD. Lysates were immunoprecipitated with control, anti-acetyl histone H3, anti-acetyl H3K18, or anti-HDAC1 antibody. Associated DNA was isolated and analyzed via real time PCR using primers spanning the RGS10-1 and GAPDH promoters. Real-time PCR values were normalized to the total amount of promoter DNA added (input). Input values represent 5% of the total cell lysate. * P<0.05. **A.** Global levels of Histone H3 acetylation associated with RGS10 and GAPDH promoters in A2780 and A2780-AD ovarian cancer cells. Values represent mean ± SEM of four independent experiments. **B.** Levels of histone H3 acetylated at lysine 18 associated with RGS10-1 and GAPDH promoters in A2780 and A2780-AD ovarian cancer cells. Values represent mean ± SEM of four independent experiments. **C.** Levels of HDAC1 associated with RGS10 and GAPDH promoters in A2780 and A2780-AD ovarian cancer cells. Values represent mean ± SEM of three independent experiments. **D.** Western blot analysis of global HDAC1 levels in A2780 and A2780-AD cells.

We next assessed histone modifications at RGS10-1 promoters using chromatin immunoprecipitation (ChIP) experiments. We compared acetylation at histones associated with the RGS10-1 promoter in A2780 and A2780-AD cells, using the GAPDH promoter as a control. Total H3 histone binding was similar at RGS10-1 and GAPDH promoters (data not shown). In contrast, acetylated H3 histone levels were significantly lower at RGS10-1 promoters in the chemoresistant A2780-AD cells, while similar levels of acetylated histone H3 were associated with the GAPDH promoter in both cell types (Figure 6A). Reduced acetylation at Lysine residue 18 in histone 3 (H3K18) is associated with cancer recurrence and poorer clinical outcome in lung, kidney, and breast cancer patients [20],[33]. To determine if loss of acetylation of this residue contributed to the loss of histone acetylation in RGS10 promoters in chemoresistant cells, we performed ChIP assays with H3K18-specific antibodies. We observed a slight but significant decrease in H3K18 association with the RGS10-1 promoter in chemoresistant cells as compared to A2780 parental cells, with no change at the GAPDH control promoter (Figure 6B).

Histone acetylation is dynamically regulated in cells by the opposing actions of histone acetyltransferases (HATs) that add the acetyl functional group to histones, and histone deacetylases (HDACs) that remove them. Class I HDACs are over expressed in ovarian cancer tissues and are thought to play a significant role in gene silencing during ovarian cancer progression [23]. We observed a striking increase in HDAC1 association with RGS10-1 promoters in A2780-AD cells as compared to parental A2780 cells. This increase reflects a specific recruitment to the RGS10-1 promoter, as HDAC1 association with GAPDH promoters was unchanged between cell lines (Figure 6C), and total HDAC1 expression levels were not higher in A2780-AD cells (Figure 6D).

**Figure 7:**
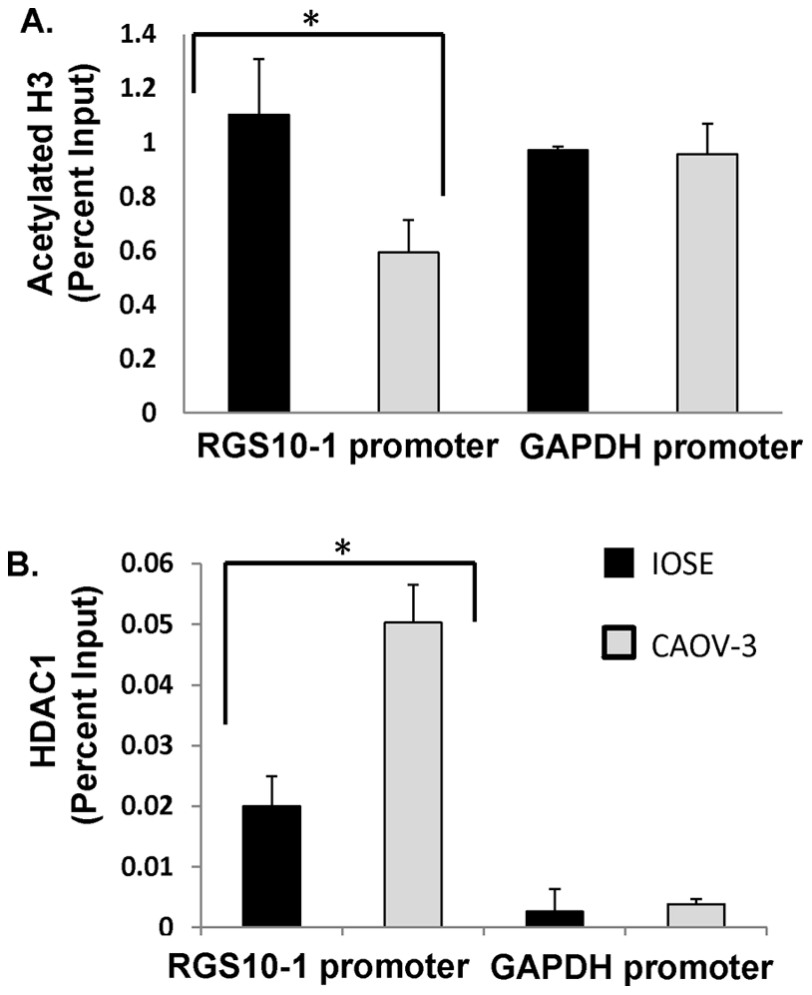
Histone acetylation and HDAC binding at RGS10-1 promoters in IOSE and CAOV-3 ovarian cells. ChIP assays were carried out in normal ovarian IOSE-80 cells and in CAOV-3 ovarian cancer cells. Lysates were immunoprecipitated with control antibody, anti-acetyl histone H3 antibody, or with anti-HDAC1 antibody. Associated DNA was isolated and quantified via real time PCR using primers spanning the RGS10 and GAPDH promoters. Real-time PCR values were normalized to the total amount of promoter DNA added (input). Input values represent 5% of the total cell lysate. * P<0.05 **A.** Global levels of Histone H3 acetylation associated with RGS10 and GAPDH promoters in normal and chemosensitive ovarian cancer cells. Values for histone H3 acetylation represent mean ± SEM of two independent experiments. **B.** HDAC1 levels associated with RGS10 and GAPDH promoters in normal and chemosensitive ovarian cancer cells. Values for HDAC1 binding are representative data. Error bars show deviation between technical errors.

To determine if histone modifications at RGS10-1 promoters may account for the difference in expression in IOSE and CAOV-3 cells, we performed ChIP assays to compare histone acetylation. Again, total histone H3 levels at the RGS10-1 promoter were unchanged between the cell lines, while the level of acetylated histone H3 associated with the RGS10-1 promoter in CAOV-3 cancer cells was half that observed in IOSE normal ovarian epithelial cells (Figure 7A). Finally, we compared the association of HDAC1 with RGS10-1 promoters in IOSE and CAOV-3 cells. The level of HDAC1 associated with the control promoter GAPDH was unchanged between cell lines, but was more than doubled at RGS10-1 promoters in the cancer cell line, compared to IOSE cells (Figure 7B). These data show that decreased RGS10-1 expression in CAOV-3 ovarian cancer cells correlates with enhanced HADC1 binding and loss of histone acetylation at the at the RGS10-1 promoter as compared to IOSE cells.

### Effects Of Hdac And Dnmt Inhibitors On A2780-Ad Cell Viability And Rgs10 Expression

**Figure 8:**
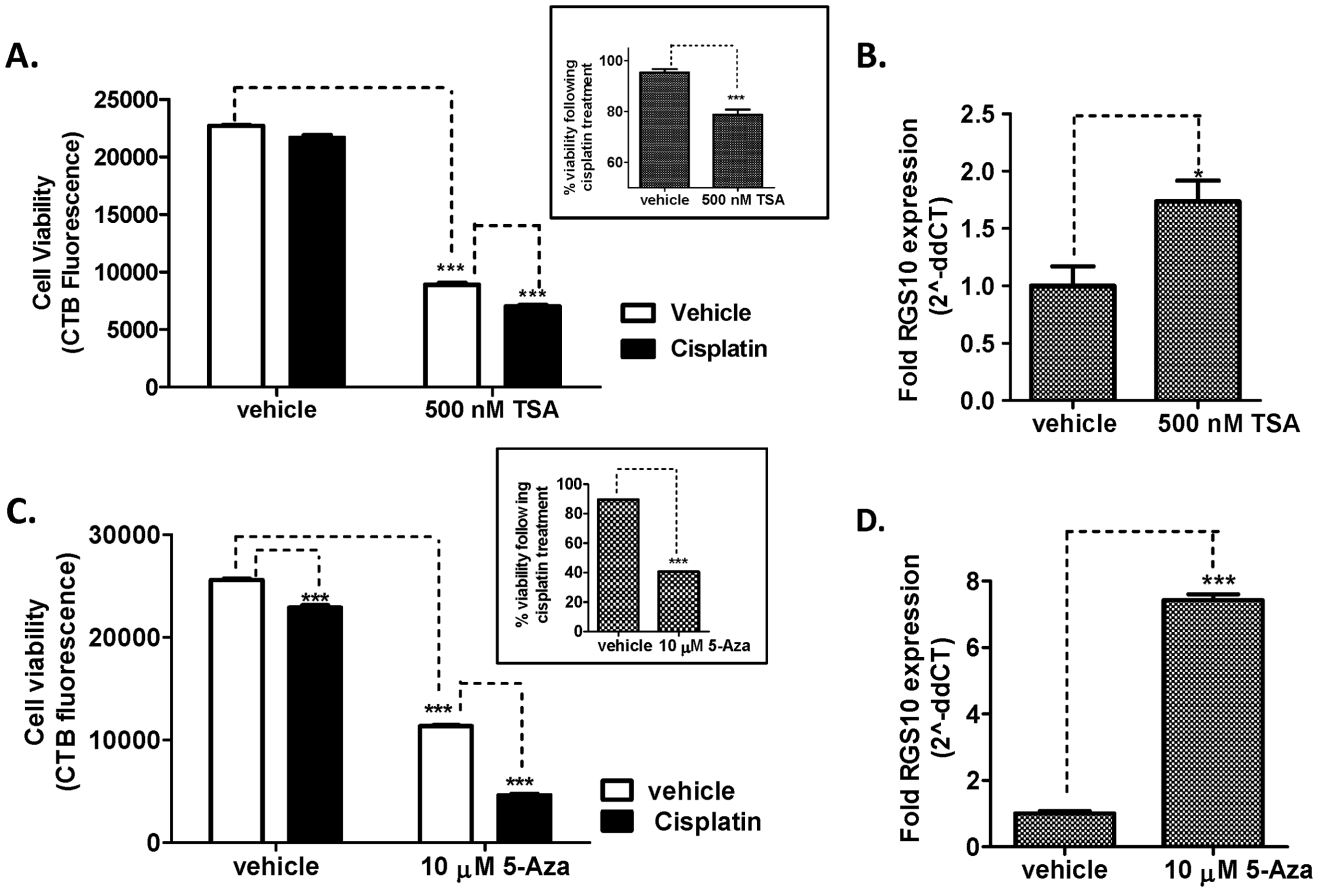
Inhibition of HDAC and DNMT enzymes in chemoresistant cells enhances cisplatin sensitivity and RGS10 expression. **A.** A2780-AD cells were plated in 96 well plates and treated with 500 nM trichostatin A (TSA) or vehicle for 48 hours, with or without 30 µM cisplatin for the final 12 hours. Cell survival was assessed using CellTiter-Blue fluorimetric viability assays. Inset: Cell viability normalized to values in the absence of cisplatin. **B.** A2780-AD cells were treated with vehicle or 500 nM TSA for 36 hours. Gene expression was assessed with RT-PCR as described, and normalized to RPL-13A gene expression. **C.** A2780-AD cells were treated with 10 µM 5 Azacytidine (5-Aza) or vehicle for 5 days, with or without 30 µM cisplatin for the final 24 hours. **D.** RGS10 mRNA expression was assessed following 5 days of vehicle or 5-Aza. ***: p<0.0001, *: p<0.05.

The previous experiments suggest that histone deacetylation may contribute to loss of RGS10 expression in chemoresistant A2780-AD cells. We next directly determined the effect of pharmacological inhibition of HDAC enzymes on A2780-AD cell viability and RGS10 expression. A2780-AD cells were grown in the presence of vehicle or 500 nM Trichostatin A (TSA) for the 48 hours, with or without the addition of 30 µM cisplatin for the final 12 hours. Cell viability was assessed at the end of the 48 hour treatment. Consistent with previous observations, cisplatin alone had minimal effect on the viability of the chemoresistant cells at this time point. The addition of TSA alone to inhibit HDAC enzymes significantly reduced cell viability. Further, following pre-treatment with TSA, the addition of cisplatin resulted in a 25% loss of cell-viability (Figure 8A). In a parallel experiment, we assessed RGS10 expression following 36 hours of vehicle or TSA treatment, corresponding to the start of the cisplatin treatment period. Inhibition of HDAC enzymes nearly doubled RGS10 transcript levels in A2780-AD cells (Figure 8B). Similar fold changes in RGS10 expression were observed following siRNA knock down of HDAC1 in A2780-AD cells. These results further suggest that HDAC-mediated silencing of RGS10 correlates with chemoresistance in ovarian cancer cells. Finally, we determined the effect of DNMT inhibition on RGS10 expression and cisplatin sensitivity in A2780-AD cells. Cells were grown in 5-Aza for five days, and cisplatin was added for the final 24 hours. 5-Aza treatment significantly enhanced cisplatin-mediated cell death from 10% to 60%, relative to controls lacking cisplatin (Figure 8C, inset), and increased RGS10 expression dramatically. Notably, the mechanism of action of 5-Aza requires longer cell incubation times than does TSA, and may account for the greater magnitude effects on cell viability and RGS10 expression of 5-Aza as compared to TSA.

## Discussion

G-proteins are an important class of signal mediators, critical in the regulation of basic function of the nervous system, cardiovascular system, immune system, and malignancies [34]. The essential mechanism by which G-proteins are activated to initiate these events is by ligand binding to G-protein coupled receptors (GPCRs). G-proteins are negatively regulated by cellular RGS proteins, which deactivate G-proteins through their GTPase activating protein (GAP) activity [35]. Therefore, the strength of G-protein signaling cascades is determined by the balance of activity of GPCRs and RGS proteins, requiring that both GPCRs and RGS proteins be tightly regulated. In the case of GPCRs, activity is controlled by binding of endogenous ligands to the extracellular surface of the receptors. Growing evidence suggests that RGS activity is regulated by multiple mechanisms controlling the expression and localization of RGS proteins. The current study marks the first description of the intricate regulation of expression of an RGS gene by histone deacetylation and DNA methylation, and establishes epigenetics modification as an additional mechanism by which RGS expression­–and indirectly G-protein activity – is regulated.

We have previously reported that expression of RGS10, which normally suppresses growth and survival signaling pathways triggered by G-protein coupled receptors, is suppressed as ovarian cancer cells develop chemoresistance [10]. This suppression indirectly amplifies G-protein mediated cell growth and survival signaling and contributes to chemoresistance. In the current study we analyzed expression of RGS10 isoforms in normal and cancer-derived ovarian cells and determined the changes in epigenetic marks on RGS10 promoter DNA and histones in cells with different RGS10 expression levels. To probe the mechanisms responsible for suppressing RGS10 expression, we focused on two comparisons. First, we compared IOSE immortalized ovarian surface epithelial cells versus CAOV-3 ovarian cancer cells, as these cells displayed the greatest fold difference in RGS10 expression. However, because these cells are derived from two different patients, the difference in RGS10 expression may represent multiple differences in the epigenetic and transcriptional machinery. Thus, we also compared A2780 and A2780-AD cells. While the change in RGS10 expression is relatively modest between these two cell lines, the fact that they are a parent-daughter cell line pair with common genetic background suggests this model may reveal more subtle and acute modifications to the RGS10 gene and its regulation.

We predicted that the RGS10-1 promoter may be epigenetically regulated by DNA methylation for multiple reasons. First, silencing of tumor suppressors via DNA hypermethylation of their promoter regions is a major mechanism for cancer progression in general [36],[37]. Second, DNA methylation is implicated in ovarian cancer chemoresistance, as global DNA methylation and DNA methyl transferase expression are both increased in cisplatin resistant A2780 ovarian cancer cells [31]. Further, inhibitors of DNA methylation re-sensitize previously resistant ovarian cancer cells to cisplatin [38]. Third, a recent report by Tu *et al*. reported epigenetic silencing of RGS2 in prostate cancer cells by promoter DNA methylation [39]. Our findings that the promoter of RGS10-1 was distinctly enriched in CpG dinucleotides and that inhibition of DNMT activity dramatically increased RGS10-1 expression supports the hypothesis that RGS10-1 transcription may be negatively regulated by DNA methylation. Further, we observed an increase in the methylation frequency of the RGS10-1 promoter in A2780-AD cells compared to parental cells, which was most prominent in a region approximately 800 basepairs upstream of the transcriptional start site. Recently released ENCODE transcription factor ChIP-Seq datasets suggest HEY-1 and c-myc transcription factors may interact with this region, as well as possible Pol-2 and Pol3 interactions [40]. Additional studies are needed to define the specific contribution of this region to the regulation of RGS10-1 expression in ovarian cancer cells and in clinical chemoresistance. In contrast, no change in RGS10-1 promoter methylation was observed between IOSE and CAOV-3 cells, suggesting that this mechanism may specifically correlate to loss of RGS10-1 expression in acquired chemoresistance.

Our results clearly demonstrate loss of histone acetylation and gain of HDAC-1 binding at RGS10-1 promoters in ovarian cancer cells with low RGS10-1 expression. This result is consistent with abundant evidence that acetylation of lysine residues in the N-terminal tails of histones H3 and H4 is frequently reduced in cancers [19],[20]. Further, Class I HDACs 1–3 are overexpressed in ovarian cancer tissues [23], and aberrant HDAC expression is associated with poor responses to chemotherapy [41]. HDAC inhibitors can inhibit cancer cell growth *in vitro* and *in vivo*, revert oncogene-transformed cell morphology, induce apoptosis, and enhance cell differentiation [42],[43]. The class I selective HDAC inhibitor romidepsin (FK228) is effective in reducing ovarian cancer cell proliferation at nanomolar concentrations [44], and multiple HDAC inhibitors are in ongoing cancer clinical trials [45],[46].

Given that RGS10 downregulation correlates with acquired chemoresistance and RGS10 knock-down directly enhances cell growth and survival, it is possible that enhancing RGS10 expression will have therapeutic benefit. Our results suggest that DNMT and HDAC enzymes may suppress RGS10 expression in ovarian cancer cells, and therefore inhibition of DNMT and HDAC enzymes should enhance RGS10 expression. HDAC inhibitors induce apoptosis in chemoresistant ovarian cancer cells [47], and DNMT inhibitors can re-sensitize chemoresistant ovarian cancer cells to cisplatin [48]. Future studies will determine if HDAC inhibition and DNMT inhibition can synergistically increase RGS10-1 expression, and define the role that RGS10 expression may play in the therapeutic effects of HDAC and DNMT inhibitors in ovarian cancer. Further, future studies should further define differences in RGS10 expression in normal ovarian and fallopian tissues and ovarian tumors. Our studies used the established IOSE cell line as a control. While these cells provide a useful control for comparison to other immortalized cell lines, it should be noted that they are an immortalized cell line and are not “normal” ovarian tissue.

Ovarian cancer is a heterogeneous disease, representing multiple cellular strategies for evading normal quiescence and apoptotic signals. Defining unique molecular signatures for populations of ovarian cancer cells with distinct growth and survival features may lead to diagnostic tools to predict drug responsiveness. Loss of RGS10 is not a universal feature of ovarian cancer cells; for example, while RGS10 expression is dramatically suppressed in CAOV-3 cells, it is not significantly suppressed in SKOV-3 serous epithelial ovarian cancer cells (not shown). We propose that loss of RGS expression may define a subclass of ovarian cancer cells that have enhanced sensitivity to G-protein coupled growth and survival signals. Determining the epigenetic status of RGS genes in individual patient tumors may lead to an (epi)genetic biomarker for tumors with resistance to traditional chemotherapy, but with enhanced sensitivity to GPCR-blocking drugs, such as LPA receptor antagonists. Finally, while our studies have focused on RGS10-1 suppression in ovarian cancer, our results have broader implications. RGS10 GAP activity selectively targets Gi-family G-proteins, and the receptors for LPA, endothelin, and SDF-1 all strongly couple to Gi proteins to mediate growth and survival responses in multiple cancers [49]–[52]. This suggests that RGS10 expression may suppress cancer cell growth and survival in a variety of tumors. Additional work is needed to determine if the epigenetic marks described here contribute to regulation of RGS10 expression in other cancers.

## Supporting Information

Figure S1 **Lollipop representation of DNA methylation in individual sequenced clones of bisulfite treated genomic DNA from the indicated cell lines.** Sequences were subjected to quality control analysis and aligned using BiQ Analyzer software. **A.** Primer region BS10-1 (−121,303,236 → −121,303,086). **B.** Primer region BS10-2 (−121,303,076 → −121,302,726). **C.** Primer region BS10-3 (−121,302,800 → −121,302,514). **D.** Primer region BS10-4 (−121,302,327 → −121,301,988). (PDF)

Table S1 **Summary of methylation frequencies in the RGS10 promoter.**
(TIF)
