# Supplementary material for: Transcriptional Suppression, DNA Methylation, and Histone Deacetylation of the Regulator of G-Protein Signaling 10 (RGS10) Gene in Ovarian Cancer Cells
Source: PLoS One. 2013 Mar 22;8(3):e60185. doi: 10.1371/journal.pone.0060185 (PMC3606337; doi:10.1371/journal.pone.0060185)
Supplement: Figure S1 — (PDF) [file pone.0060185.s001.pdf]

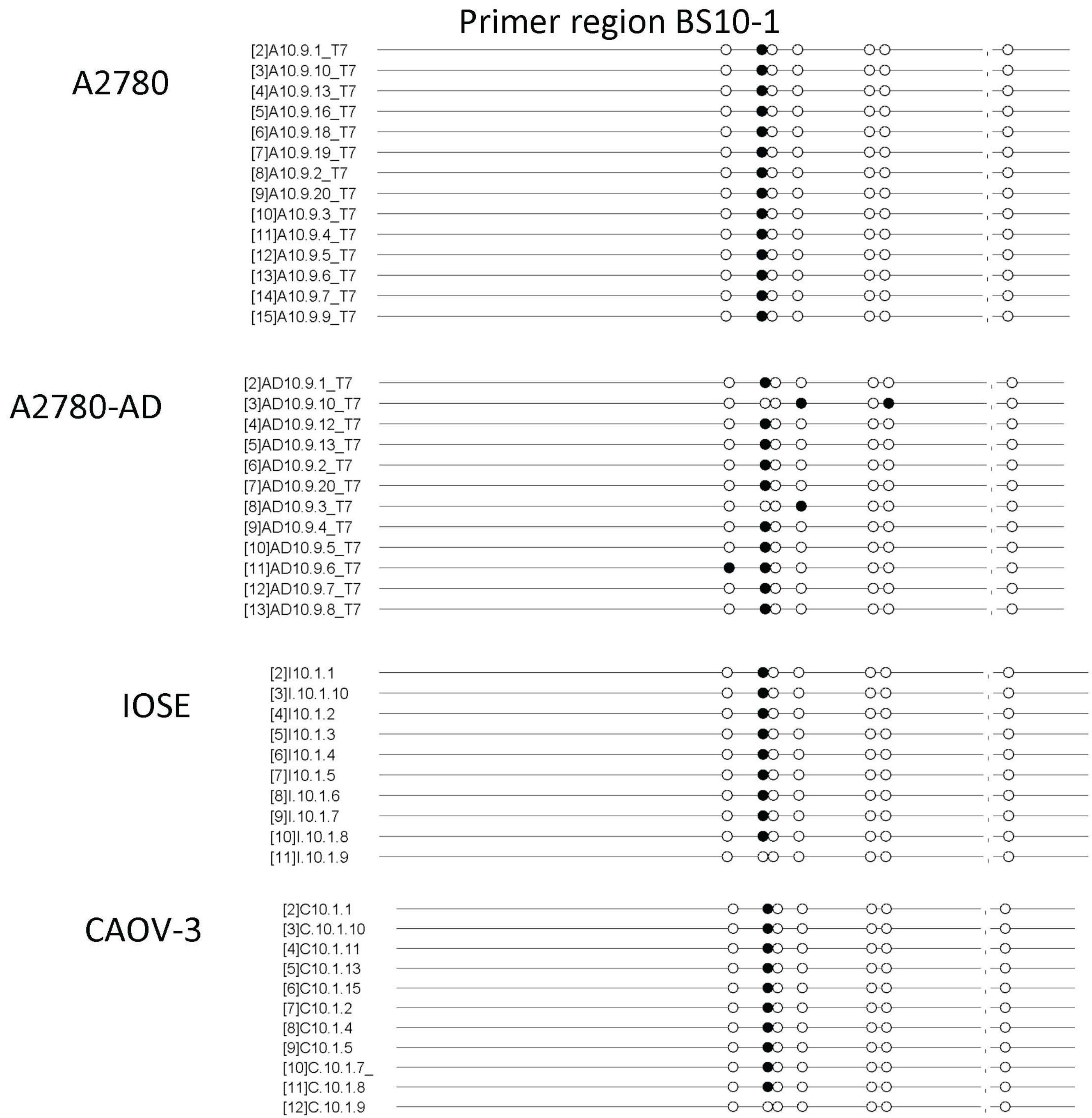

Supplementary data 1A

Primer region BS10-2

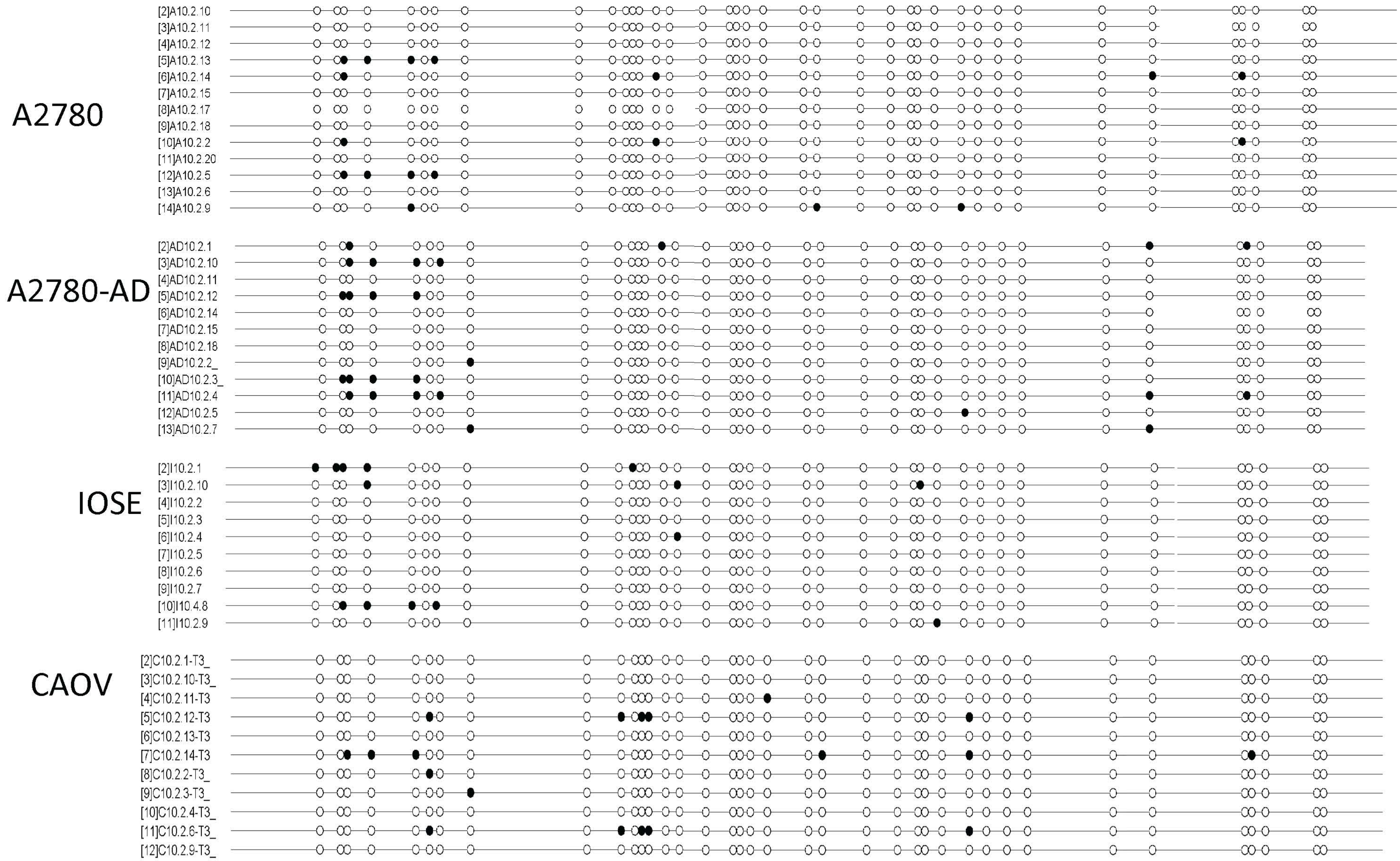

Supplementary 1B

Primer region BS10-3

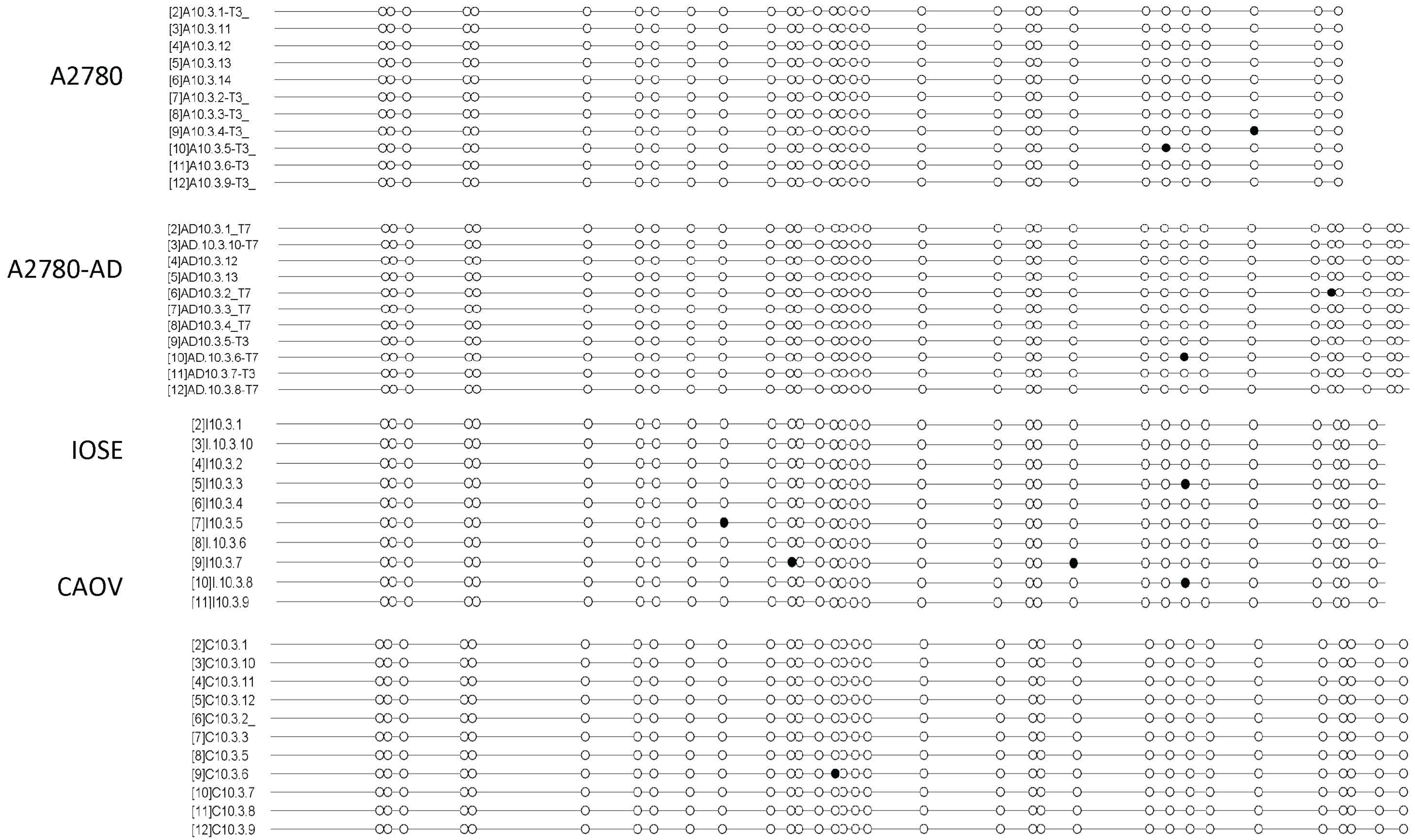

Supplementary 1C

Primer region BS10-4

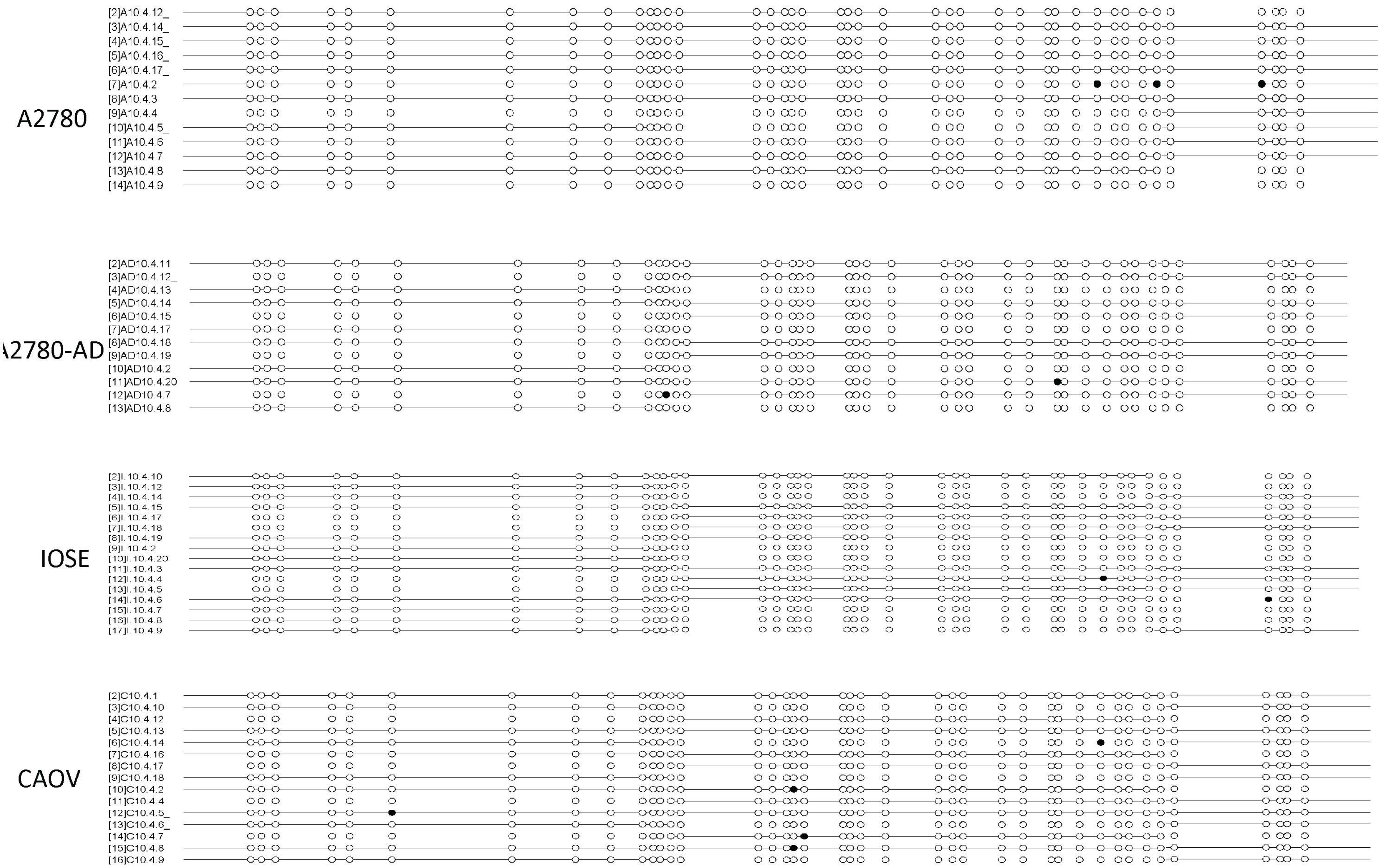

Supplementary 1D
