# Supplementary figures and images for: Transcriptional Suppression, DNA Methylation, and Histone Deacetylation of the Regulator of G-Protein Signaling 10 (RGS10) Gene in Ovarian Cancer Cells
Source: PLoS One. 2013 Mar 22;8(3):e60185. doi: 10.1371/journal.pone.0060185 (PMC3606337; doi:10.1371/journal.pone.0060185)

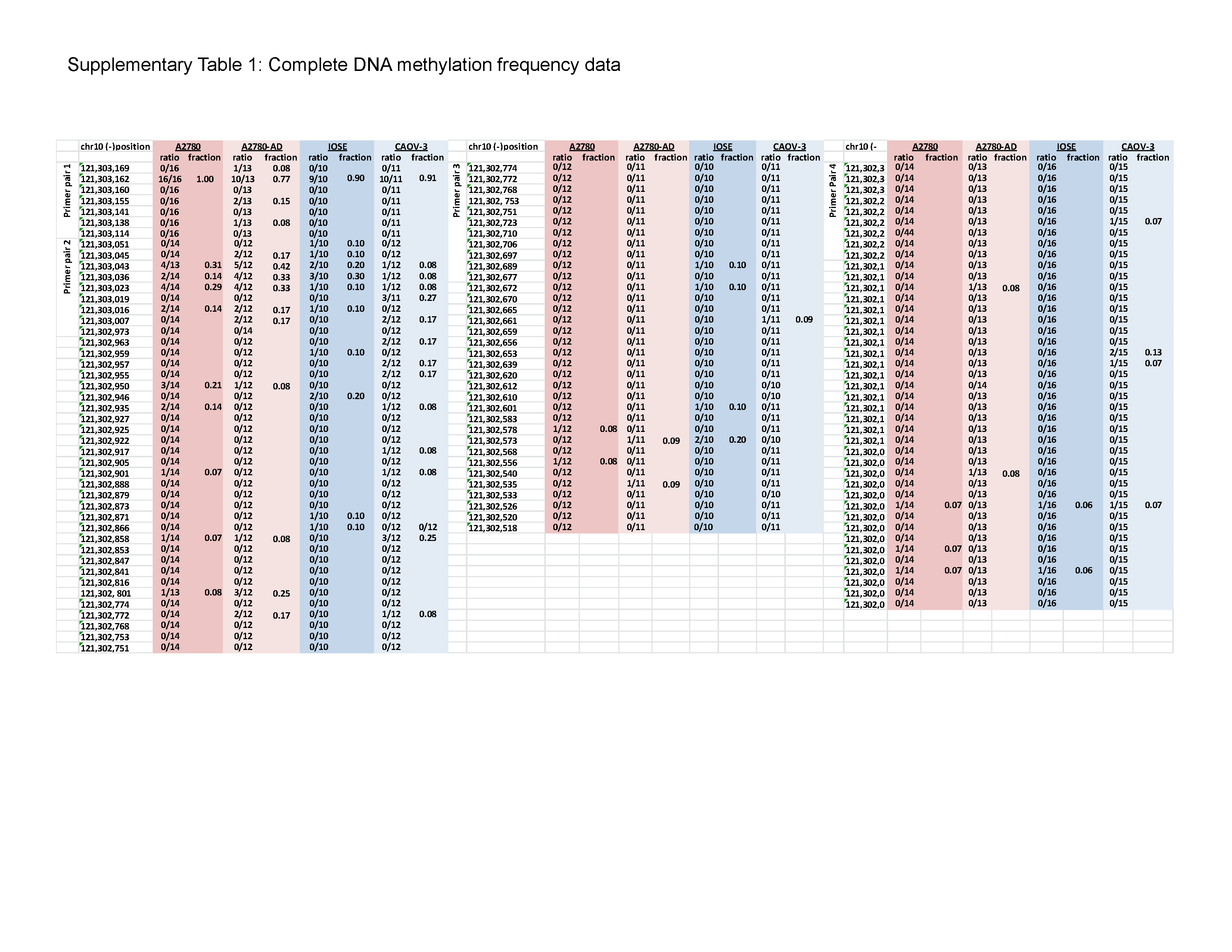

Supplement: Table S1 — (TIF) [file pone.0060185.s002.tif]
